# Supplementary material for: Splice-Junction-Based Mapping of Alternative Isoforms in the Human Proteome
Source: Cell Rep. Author manuscript; Available in PMC 2020 Jan 15. (PMC6961840; doi:10.1016/j.celrep.2019.11.026)
Supplement: 2 [file NIHMS1546469-supplement-2.zip › DF1/PXD006675/RightAtrium/RightAtrium_38_HMGB1_ENILACPLVMLR.pdf]

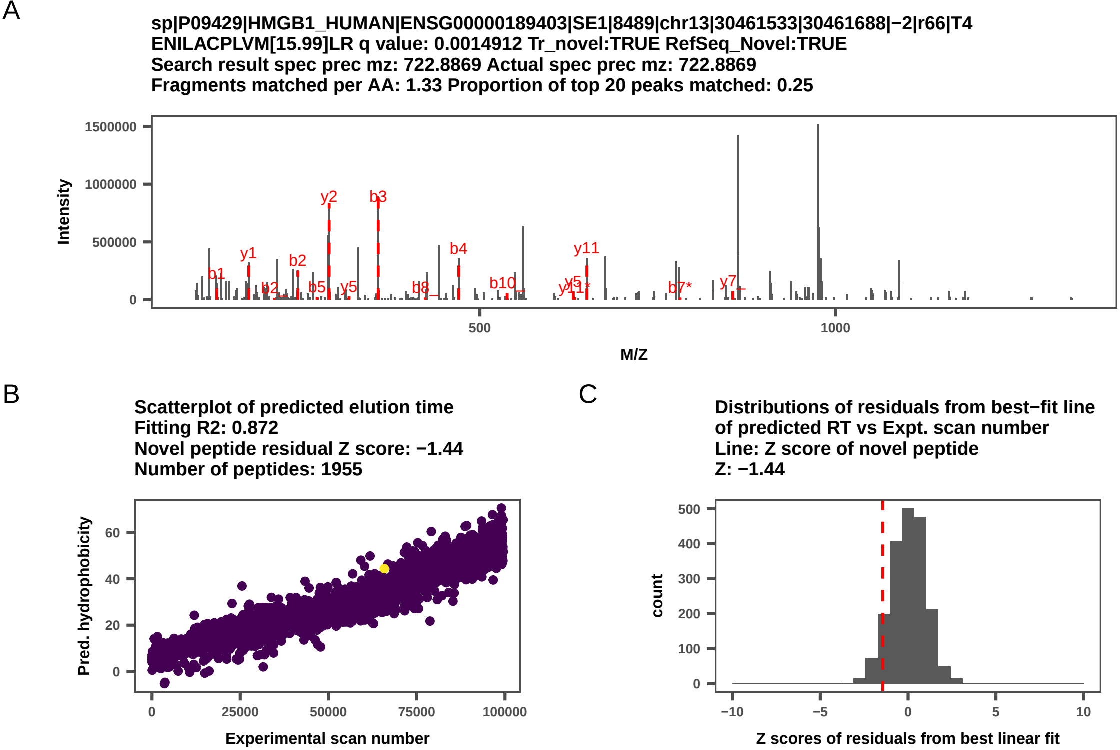
A
sp|P09429|HMGB1_HUMAN|ENSG00000189403|SE1|8489|chr13|30461533|30461688|−2|r66|T4
ENILACPLVM[15.99]LR q value: 0.0014912 Tr_novel:TRUE RefSeq_Novel:TRUE
Search result spec prec mz: 722.8869 Actual spec prec mz: 722.8869
Fragments matched per AA: 1.33 Proportion of top 20 peaks matched: 0.25
Intensity
1500000
1000000
500000
0
y2
b3
y1
b2
b1
b4
y11
b10
y5
y11*
b7*
y7
b5
b8
500
1000
M/Z
B
Scatterplot of predicted elution time
Fitting R2: 0.872
Novel peptide residual Z score: −1.44
Number of peptides: 1955
Pred. hydrophobicity
60
40
20
0
0
25000
50000
75000
100000
Experimental scan number
C
Distributions of residuals from best−fit line
of predicted RT vs Expt. scan number
Line: Z score of novel peptide
Z: −1.44
count
500
400
300
200
100
0
−10
−5
0
5
10
Z scores of residuals from best linear fit
